# Comparison of the COBAS/Ampliprep Taqman and Amplicor HIV-1 monitor tests in Lagos, Nigeria

**DOI:** 10.4102/ajlm.v2i1.68

**Published:** 2013-05-15

**Authors:** Olufemi S. Amoo, Idowu A. Taiwo, Olumuyiwa O. Salu, Azuka P. Okwuraiwe, Chika K. Onwuamah, Morenike A. Awe, Osaga O. Oforomeh, Daniel I. Onwujekwe, Oliver C. Ezechi, Audu R. Ajuma

**Affiliations:** 1 Human Virology Laboratory, Nigerian Institute of Medical Research, Yaba, Lagos, Nigeria; 2 Department of Cell Biology & Genetics, University of Lagos, Akoka, Lagos, Nigeria; 3 Clinical Sciences Division, Nigerian Institute of Medical Research, Yaba, Lagos, Nigeria

## Abstract

**Background:**

The use of real-time Polymerase chain reaction (PCR) technology options is increasing in resource-limited settings because they are faster, improve assay sensitivity, have higher throughput, larger dynamic ranges and reduced rates of contamination. In 2010, UNAIDS ranked Nigeria as the second highest population of people living with HIV and AIDS (2.98 million people) in the world.

**Objective:**

The objective of this study was to compare the analytical performances of the Amplicor HIV-1 Monitor (version 1.5) and the COBAS Ampliprep/Taqman (version 2.0) used in monitoring HIV disease progression in HIV-infected individuals.

**Method:**

In a cross-sectional study, HIV-1 RNA values obtained with the Amplicor HIV-1 monitor version 1.5 were compared with those of the COBAS/Ampliprep TaqMan HIV-1 version 2.0 in a routine clinical setting. Between May and November 2011, 176 plasma samples collected were analysed in parallel using both techniques. Data analysis was done using statgraphics Centurion XVI and Medcalc version 12.0.

**Result:**

The correlation coefficient for the two assays was 0.83 and the level of agreement using a Bland–Altman plot was 94.2%.

**Conclusion:**

These findings suggest that the results from the two methods were comparable, hence the COBAS/Ampliprep Taqman version 2.0 is recommended for high-volume laboratories.

## Introduction

The World Health Organisation guidelines for the treatment of HIV-1-infected patients recommend viral load as a major marker in disease prognosis.^1^ In conjunction with other immunological tests, HIV viral load is used to assess the efficacy of antiretroviral drugs. Therefore, accurate measurement of HIV-1 viral load is essential to provide clinicians with valuable information to determine treatment decisions. Recently, new quantitative HIV-1 assays have been designed to cope with increasing molecular diversity of the virus, to overcome the issue of turnaround time and the challenges of viral load estimation encountered with manual methods.^2^ However, there have been reports of plasma viral load discrepancies between the Amplicor monitor test and one of the technologically improved methods, the COBAS Ampliprep/Taqman.^3^ In a study in South Africa, both assays have been reported to have a good agreement,^4^ and it is important that a similar study is repeated because of the different subtypes found in this region. Therefore, there is need to establish a relationship between these two assays.

Manual methods for nucleic acid extraction are the most time-consuming and challenging aspect of viral load measurement. In addition, they require skilled technical personnel and extended ‘hands-on’ time. Automation of the extraction process, on the other hand, has the potential to significantly increase reliability, sample throughput and efficiency. Globally, Nigeria has the second highest number of people infected with HIV.^5^ The widespread use of antiretroviral drugs and their availability in low-resource countries has not only brought a form of relief by improving the health of the individuals infected with HIV but has also led to more people living with HIV and AIDS seeking care and treatment.^6^ In Nigeria, assays are being expanded to manage more patients because of the evidence of their use in early detection of drug resistance.^7^ Therefore, there is need for testing laboratories to prepare to meet with the high demand as it has to do with meeting turnaround time and providing the quality of results needed for efficient patient management. Due to the superior technology and ease of use of the COBAS Ampliprep/Taqman, it is recommended that it replace the manual Amplicor as a monitoring tool for HIV-1 RNA. However, it is good laboratory practice that these monitoring tools are validated before use, especially in places where various HIV-1 subtypes exist.^8,9,10^ The aim of this study was therefore to compare HIV-1 RNA values obtained with the Amplicor HIV-1 monitor version 1.5 with those of the COBAS TaqMan HIV-1 assay in a routine clinical setting.

## Materials and method

In a retrospective study between May and November 2011, 176 archived plasma samples previously tested with the Amplicor monitor test and stored at −80 °C in the Human Virology Laboratory were assayed for HIV-1 viral load using the Amplicor monitor version 1.5 HIV-1 viral load technique. Samples within the detection range of 400 copies/mL and 750 000 copies/mL were selected and assayed with the COBAS Ampliprep/Taqman version 2.0. The subjects’ informed consent was obtained before inclusion in the study. Data were analysed using Epi Info 2008 (version 3.5.1), STATGRAPHICS Centurion XVI (version 16.0.3) and Microsoft Office Excel 2007. The results are presented as mean and standard deviation (s.d.). Agreement between the two methods being compared was also assessed using correlation coefficient, linear regression and Bland-Altman analysis. Differences between means were considered significant when *P* ≤ 0.05.

## Viral load assay

**Amplicor HIV-1 monitor test (version 1.5):** This assay targets only the gag p24 region using conventional Polymerase chain reaction (PCR) method. The lower limit of quantitation is 2.60 log_10_ copies/mL and upper limit of quantitation is 5.87 log_10_ copies/mL. The standard specimen volume is 200 μL. Nucleic acid were extracted, amplified and hybridised as recommended by the kit manufacturer. The Amplicor HIV-1 monitor test is based on five major processes, namely specimen preparation; reverse transcription of target RNA to generate complimentary DNA (cDNA); PCR amplification of target cDNA using HIV-1 specific complementary primers; hybridisation of the amplified products to oligonucleotide probes specific to the targets; and detection of the probe linked with an enzyme so as to give color reaction later on.

**Cobas AmpliPrep/Cobas TaqMan HIV-1 test, version 2.0:** This assay simultaneously targets the gag and the LTR region with two dually labelled hybridisation probes. The lower limit of quantitation is 1.30 log_10_ copies/mL and upper limit of quantitation is 7.0 log_10_ copies/mL. The specimen volume required for this method is 1000 μL. Upon loading the sample in appropriate racks, nucleic acid extraction, amplification and detection are performed using the COBAS TaqMan HIV-1 Test, v2.0 software on the COBAS AmpliPrep/COBAS TaqMan instrument as specified by equipment manual. The COBAS AmpliPrep/COBAS TaqMan HIV-1 Test is based on three major processes: specimen preparation to isolate HIV-1 RNA; reverse transcription of the target RNA to generate cDNA; and simultaneous PCR amplification of target cDNA and detection of cleaved dual-labeled oligonucleotide probes specific to the target.

**Sequencing and subtyping:** The Viroseq kit and ABI 3130xl Genetic Analyser were used to sequence the samples. Sequences for reference subtypes and CRFs were downloaded from Los Alamos sites; they were aligned and bootstrapped with patients’ sequences in ClustalX and visualised NJ plots.

## Ethical considerations

Ethical approval has been reviewed; the protocol and safety guidelines satisfied the conditions of the Nigerian Institute of Medical Research (NIMR) Institutional Review Board (IRB), policies regarding experiments that use human subjects.

## Results

**FIGURE 1:**
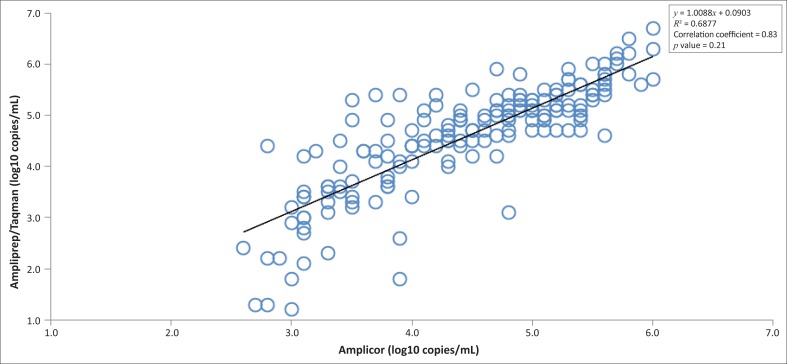
The correlation coefficient between the COBAS Ampliprep/Taqman HIV-1 assay and the COBAS Amplicor assay.

**FIGURE 2:**
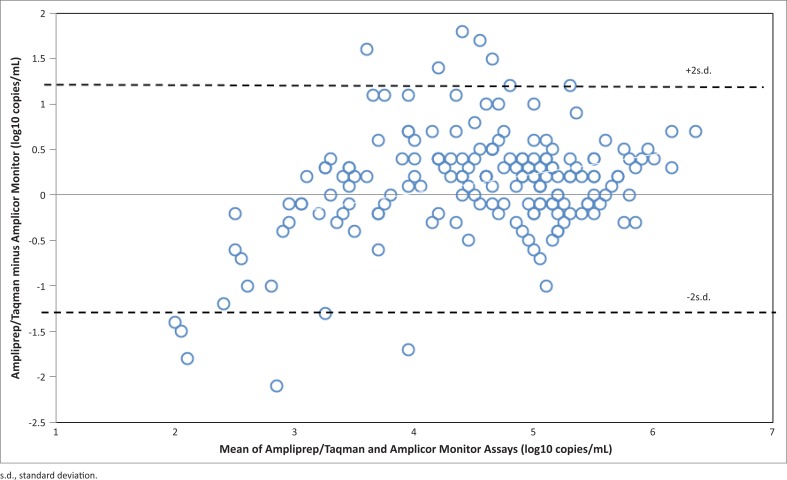
A Bland-Altman plot showing the degree of agreement between the COBAS Ampliprep/Taqman and the COBAS Amplicor monitor assays. The numbers of samples ranging within the mean ±2 s.d. interval is 163 of 176 (94.2%).

We examined 176 stored plasma specimens of HIV positive patients obtained between May and November, 2011. Analysis of the 176 samples in which viral load was determined revealed that discrepancies of more than 0.5 log_10_ copies/mL existed for 44 (25%) samples. Of these 44 samples with discrepancies, 29 (66%) had lower values with the Amplicor monitor test while 15 (34%) had higher values. Twenty samples were sequenced from the 176 samples and their subtypes were obtained. The correlation coefficient between the COBAS Amplicor and the Ampliprep/Taqman for these samples are shown according to subtypes obtained in Table 1. They all had good correlation coefficient between the assays, with the exception of subtype CRF 43_02G (*r* = 0.42). There was a strong correlation coefficient between the viral load values obtained with the 176 samples for the two assays (*r* = 0.83 *P* value < 0.21 Figure 1). The overall performance between the two assays also indicates a strong relationship, with 94.2% degree of agreement as revealed by the Bland–Altman graph (Figure 2), which represents the number of samples ranging within the mean ± 2 s.d. interval.

**TABLE 1:** Correlation coefficient between assays for HIV-1 Subtypes.

HIV-1 Subtypes	Number of samples	Correlation coefficient (*r*)	Linear regression equation *Y* = *ax* + *b*
CRF 02_AG	10	0.83	1.2442*x* - 1.4621
CRF 43_02G	5	0.42	0.4308*x* + 2.8528
G	3	0.99	0.7895*x* + 0.9632
CRF 06_cpx	2	1	4.6*x* - 15.34
All subtypes	20	0.73	1.0513*x* – 0.3838

## Discussion

Generally, there was a good correlation coefficient in viral loads between the assays for all subtypes except CRF 43_02G. However, in Nigeria, the subtypes AG and G are most prevalent^15^ and the viral loads for these subtypes were well correlated in this study.^8^ Of the 44 samples with discrepancies of more than 0.5 log_10_ copies/mL, the Amplicor Monitor assay had more samples with lower viral load titre values compared to the COBAS Ampliprep/Taqman. The COBAS Ampliprep/Taqman targets both the gag and LTR region, whereas the Amplicor Monitor assay targets only the gag region.

Overall, these data indicate that the two assays have similar performances for the quantitation of HIV-1 RNA amongst the samples tested. The high level of agreement (94.2%) observed between the two assays in this study was also reported by previous authors^12,13^ who demonstrated good overall agreement of the test results using absolute bias plot.^14^ Recently, in a study to investigate the impact of genetically diverse HIV samples from China on performance of COBAS Ampliprep/Taqman and Amplicor Monitor assays, it was demonstrated that the viral loads of different HIV genotypes measured by the use of the two assays were comparable.^11^

The COBAS Ampliprep/Taqman HIV-1 version 2.0 assay is a fully automated system with continuous sample loading and thereby has higher throughput, shorter turnaround time and minimum risk of contamination throughout sample processing. This has a major advantage for clinical laboratories in efficient patient management, especially for a laboratory such as ours that assays over 17 000 HIV-1 RNA viral load samples annually. Using the COBAS system, the laboratory could assay 36 000 viral load samples annually, with fewer skilled personnel. Moreover, the Nigerian government plans to expand access to HIV-1 viral load testing in the current ART programme, so the use of this automated system will make it easier to cope with the increased demand.

In conclusion, the study shows that there is no significant average bias between the two assays compared. However, laboratory personnel and physicians should be aware that good laboratory practice and other factors could influence the outcome of laboratory reports.
